# Immuno-Nutritional Profiling for Survival Stratification in Gastrectomized Patients with Malignant Chronic Intestinal Failure

**DOI:** 10.3390/nu18030451

**Published:** 2026-01-29

**Authors:** Konrad Matysiak, Magdalena Szewczuk, Aleksandra Hojdis, Tomasz Banasiewicz

**Affiliations:** 1 Centre for Intestinal Failure, Poznan University of Medical Sciences, 60-355 Poznań, Poland; 2 Department of Gastroenterology, Poznan University Hospital, 60-355 Poznań, Poland; magdaszewczuk@wp.pl (M.S.); aleksandra.hojdis@gmail.com (A.H.); 3 Department of General, Endocrine and Gastroenterological Oncology Surgery, Poznan University of Medical Sciences, 60-355 Poznań, Poland; tbanasiewicz@op.pl; 4 Department of Internal Medicine, Poznan University of Medical Sciences, 60-355 Poznań, Poland

**Keywords:** advanced gastric cancer, malignant intestinal failure, home parenteral nutrition, malnutrition, immuno-nutritional biomarkers, overall survival

## Abstract

**Background/Objectives**: Patients who undergo gastrectomy for gastric adenocarcinoma and subsequently develop chronic intestinal failure requiring long-term home parenteral nutrition (HPN) represent a clinically vulnerable cohort in whom survival is shaped by profound nutritional depletion and systemic inflammation. Immuno-nutritional biomarkers may support improved risk stratification in this setting. **Methods**: This retrospective study included adults who underwent gastrectomy for gastric cancer and developed malignant chronic intestinal failure requiring HPN. Immuno-nutritional status at HPN qualification was evaluated using the Controlling Nutritional Status (CONUT) score and the lymphocyte-to-monocyte ratio (LMR). Overall survival was analysed using Cox proportional hazards models. LMR discrimination was assessed using receiver operating characteristic (ROC) analysis with a Youden-derived cut-off, and differences in AUC were tested using DeLong’s method. **Results**: Ninety-seven patients met the inclusion criteria. Median overall survival was 176 days. In multivariable analysis, CONUT and LMR were the only independent predictors of survival. Each one-point increase in CONUT was associated with an approximately 70% increase in mortality risk. LMR demonstrated good discriminative ability (AUC 0.795), and a cut-off of 2.083 differentiated survival trajectories. The combined CONUT–LMR model improved prognostic classification, and DeLong’s test confirmed a significant AUC difference compared with single-marker models. Kaplan–Meier curves showed clear separation across CONUT and LMR strata (log-rank *p* < 0.001). **Conclusions**: Among patients requiring long-term HPN after gastrectomy for gastric cancer, CONUT and LMR provide complementary prognostic information. Their combined use enhances survival stratification and may support earlier identification of patients with high-risk trajectories.

## 1. Introduction

Gastric cancer remains a leading global cause of cancer-related mortality and continues to pose substantial therapeutic challenges, as many patients still present with advanced-stage disease despite progress in surgery, targeted therapies, and immuno-oncology [1,2]. Optimal management requires a coordinated multidisciplinary approach addressing oncologic, metabolic, and nutritional considerations [3]. Malnutrition is frequently present at diagnosis [4], reflecting tumor-driven catabolic and inflammatory processes [5], and is associated with impaired immune competence, reduced tolerance to systemic therapies, delayed recovery, and suboptimal postoperative outcomes [6,7].

In this context, objective, biomarker-based assessment of nutritional status, integrating metabolic, immune, and inflammatory components, has gained clinical relevance [8]. Commonly used indices include the Naples Prognostic Score (NPS) [9], the Controlling Nutritional Status (CONUT) score [10], and the Prognostic Nutritional Index (PNI) [11]. These indices have been associated, respectively, with response to neoadjuvant chemotherapy, survival and postoperative quality of life, and overall survival [12,13,14]. In parallel, inflammation and immunity related markers including the neutrophil-to-lymphocyte ratio (NLR) [15], lymphocyte-to-monocyte ratio (LMR) [16], and total lymphocyte count (TLC) [17] have demonstrated prognostic relevance across gastrointestinal malignancies, with elevated NLR and reduced LMR or TLC generally indicating worse outcomes [18,19,20]. Although these tools have been examined in surgical cohorts of gastric cancer [21,22,23], their prognostic performance among patients who develop chronic intestinal failure and require long-term home parenteral nutrition remains insufficiently understood.

We conducted a secondary, extended-cohort analysis focusing on patients who had undergone gastrectomy for gastric cancer and subsequently developed chronic intestinal failure requiring long-term home parenteral nutrition HPN. The objective of the analysis was to assess the association between selected nutritional and immune–inflammatory biomarkers and overall survival and to evaluate their prognostic value in this highly specialized clinical setting.

## 2. Materials and Methods

### 2.1. Study Population

This study is a secondary analysis based on an expanded cohort. Earlier reports included patients treated between 1 January 2015, and 31 December 2023 [24], with follow-up completed on 31 July 2024. For the present work, the accrual period was extended through 31 December 2024, and additional eligible cases were incorporated. Follow-up for all participants concluded on 31 July 2025. The analysis focuses on a predefined, clinically distinct gastric cancer subgroup: adults who developed chronic intestinal failure after gastrectomy and required long-term home parenteral nutrition (HPN). Consecutive patients meeting these criteria within the expanded accrual window were included, and vital status and clinical outcomes were ascertained through the final follow-up date.

All patients were consecutively enrolled at the time of qualification for home parenteral nutrition. During the study period, all eligible patients received palliative chemotherapy, resulting in a clinically homogeneous cohort and obviating the need for additional exclusion criteria. Palliative chemotherapy was administered outside our centre according to standard oncological practice, and treatment decisions regarding chemotherapy regimens and dosing were made independently by the treating oncologists. At the time of qualification for nutritional therapy, none of the patients were receiving therapeutic doses of systemic corticosteroids, including dexamethasone, for any indication.

Inclusion criteria were:

- Gastrectomy performed for gastric cancer according to the TNM classification.
- Chronic intestinal failure requiring parenteral nutrition. A pathophysiological mechanism of intestinal failure classified in all cases as malignant mechanical obstruction.
- Patients were additionally required to have a Karnofsky Performance Status ≥ 50 at the time of HPN qualification.

Exclusion criteria were:

- Refractory malignant ascites.
- Hepatic metastases.
- Anastomotic leakage at the esophago-jejunal anastomosis.
- Obstructive jaundice.
- Complications related to nutritional therapy.
- Lack of patient consent.

Primary objective. To identify nutritional and immune-inflammatory biomarkers associated with overall survival in patients with chronic intestinal failure following gastrectomy for advanced gastric cancer who required long-term home parenteral nutrition.

**Figure 1:**
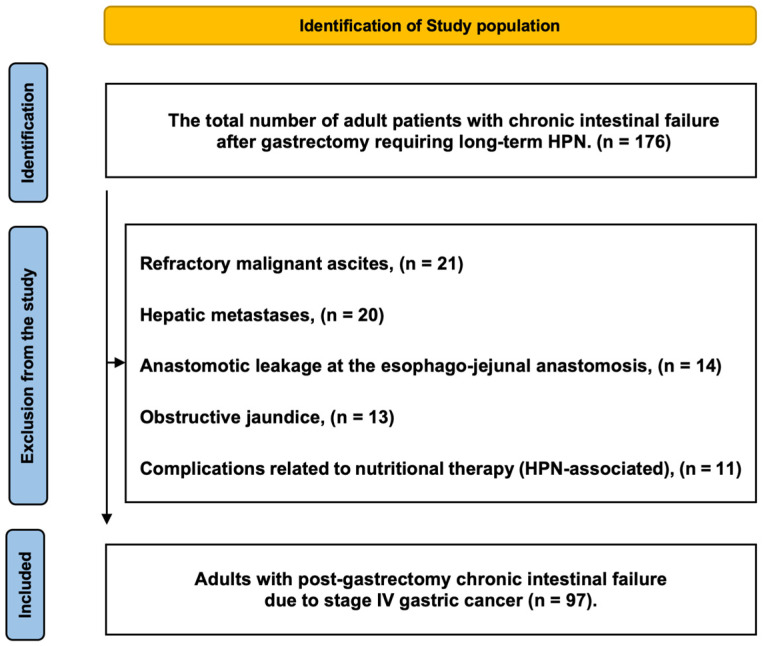
Flow diagram illustrating the selection of the study cohort and the application of predefined eligibility criteria for the final analytic sample [25].

Secondary objective. To evaluate the interrelationships among immune-nutritional indices and to characterize the strength and direction of these associations, aiming to confirm the internal biological coherence of biomarkers reflecting systemic inflammation, immune competence, and nutritional status. Figure 1 provides a detailed overview of patient identification, applied exclusion criteria, and the final cohort included in the analysis.

### 2.2. Data Collection

Nutritional status and immune–inflammatory activity were assessed from laboratory tests obtained on the day of hospital admission for qualification for HPN.

All laboratory parameters used for immuno-nutritional assessment were collected at baseline, prior to the initiation of home parenteral nutrition and palliative chemotherapy. Nutritional status and immune–inflammatory activity were assessed using laboratory tests obtained on the day of hospital admission for qualification for home parenteral nutrition. Clinical and laboratory data were complete across the analysed cohort; therefore, no imputation methods were required.

Nutritional status was evaluated using

- The Controlling Nutritional Status score, calculated from serum albumin, total cholesterol, and Total Lymphocyte Count; patients were categorized into four levels ranging from normal nutritional status to severe malnutrition [10].
- Total Lymphocyte Count was derived as the product of the total white blood cell count and the lymphocyte percentage [17].
- The Prognostic Nutritional Index was calculated from serum albumin and TLC [11].
- The Naples Prognostic Score, incorporating serum albumin, total cholesterol, neutrophil-to-lymphocyte ratio, and lymphocyte-to-monocyte ratio, was used to classify patients into low-, intermediate-, and high-risk groups [9].

Individual immune–inflammatory indices were also assessed, including the

- Lymphocyte-to-monocyte ratio [16].
- Neutrophil-to-lymphocyte ratio [15].

Body mass index (BMI) was calculated as weight (kg) divided by height squared (m^2^). All biochemical analyses were performed in a certified clinical laboratory according to applicable quality standards and Good Laboratory Practice. The test panel included complete blood count with differential (white blood cells, lymphocytes, monocytes, neutrophils) and serum total protein, albumin, and total cholesterol.

In the analysed cohort, HPN was administered according to the standard protocol applied in our institution, based on current ESPEN recommendations [26].

Nutritional support was provided using commercially premixed three-chamber parenteral nutrition admixtures, supplemented with additional fluids as clinically indicated. The premixed three-chamber bags used included Olimel or Multimel (Baxter Ltd., Deerfield, IL, USA) and Kabiven or SmofKabiven (Fresenius SE & Co., KGaA Health Care Group, Bad Homburg, Germany). These admixtures were delivered to patients, together with a standardized supplementation of trace elements and vitamins. Admixtures were additionally supplemented with electrolyte solution (Optilyte; Fresenius SE & Co., KGaA Health Care Group, Bad Homburg, Germany), in volumes ranging from 500 to 1000 mL, depending on clinical indications. In addition to home parenteral nutrition, oral intake was limited to regular food according to individual tolerance and supervised by a dietitian; no specialized oral nutrition support was provided

### 2.3. Statistical Analysis

All analyses were conducted in Python 3.12.0 using lifelines 0.29.0, scikit-learn 1.5.2, SciPy 1.14.1, statsmodels 0.14.2, matplotlib 3.9.2, and seaborn 0.13.2. All tests were two-sided.

Continuous variables, which were non-normally distributed with outliers, were summarized using medians and interquartile ranges (IQRs); minimum and maximum values were also reported. Between-group comparisons were performed using the Mann–Whitney U test. Categorical variables were described using counts and percentages and compared using the chi-square test of independence or Fisher’s exact test, as appropriate for small samples.

Overall survival was initially explored using univariable Cox proportional hazards models. Variables significant in univariable analysis were considered for multivariable Cox regression. Effect sizes were reported as hazard ratios (HRs) with 95% confidence intervals (CIs) and corresponding *p*-values. Model stability was assessed using collinearity diagnostics, including variance inflation factor (VIF) and correlation matrices; VIF < 5 was considered acceptable. The proportional hazards assumption was evaluated using Schoenfeld residuals. When violations were detected, time-varying coefficient Cox models or stratified Cox models were applied as appropriate.

Because LMR showed substantial skewness, its discriminatory performance was further evaluated using ROC curve analysis. The optimal cutoff point was determined using the Youden index and used to dichotomize LMR for confirmatory analyses. For variables retaining significance in multivariable models, Kaplan–Meier survival curves were constructed and compared using log-rank tests.

For CONUT, adjacent-category contrasts (1 vs. 2, 2 vs. 3, 3 vs. 4) were tested using the Holm correction for multiple comparisons. A Cox model treating CONUT as an ordinal variable was also fitted to assess a monotonic risk trend.

To compare the classification performance of survival prediction models, ROC analysis was conducted. For models based on single and combined markers (CONUT and CONUT + LMR), AUC values and 95% CIs were estimated using 5000 bootstrap replications. AUC differences were tested using DeLong’s method. To evaluate the incremental prognostic value of LMR, Net Reclassification Improvement (NRI) and Integrated Discrimination Improvement (IDI) indices were also calculated. A significance threshold of *p* < 0.01 was prespecified for all primary analyses. Where stricter thresholds were achieved (e.g., *p* < 0.001 in log-rank tests), these are explicitly reported. For comparative model performance assessments (AUC, DeLong test, NRI, IDI), a conventional level of *p* < 0.05 was used.

## 3. Results

### 3.1. Patient Characteristics and Survival

A total of 97 patients who underwent gastrectomy for gastric adenocarcinoma were included (47 women, 50 men; age 32–87 years). The median overall survival was 176 days (IQR, 472; range, 25–1194). All patients had chronic malignant small-bowel obstruction secondary to peritoneal metastases of adenocarcinoma. The interval from gastrectomy to HPN qualification ranged from 28 to 243 days. During HPN, all patients received concurrent 5-fluorouracil–based palliative chemotherapy.

The mean caloric intake during HPN was 27.2 ± 7.4 kcal/kg body weight, and the mean daily fluid volume was 1682 ± 460 mL. Owing to progression of intestinal obstruction, 28 patients (29%) required rehospitalization; 14 were rehospitalized twice and 8 three times. The mean length of stay per hospitalization was 8 ± 3 days.

### 3.2. Laboratory Parameters and Immunonutritional Indices

Selected laboratory parameters were compared between patients with survival shorter versus longer than the median of 176 days (Table 1).

To further evaluate nutritional status and inflammatory activity, the following indices were analyzed: NPS, CONUT, BMI, NLR, LMR, TLC, and PNI. Comparisons were performed relative to median survival. Several parameters, lymphocyte count, lymphocyte percentage, serum albumin, total protein, NPS, CONUT, LMR, TLC, and PNI, differed significantly between the two groups (Table 2).

### 3.3. Cox Regression Analysis

#### 3.3.1. Univariable Screening of Predictors

In univariable Cox models, CONUT, PNI, LMR, NPS, TLC, and NLR were significantly associated with mortality risk, whereas BMI was not and was therefore excluded from multivariable modeling (Table 3).

#### 3.3.2. Collinearity Diagnostics

**Figure 2:**
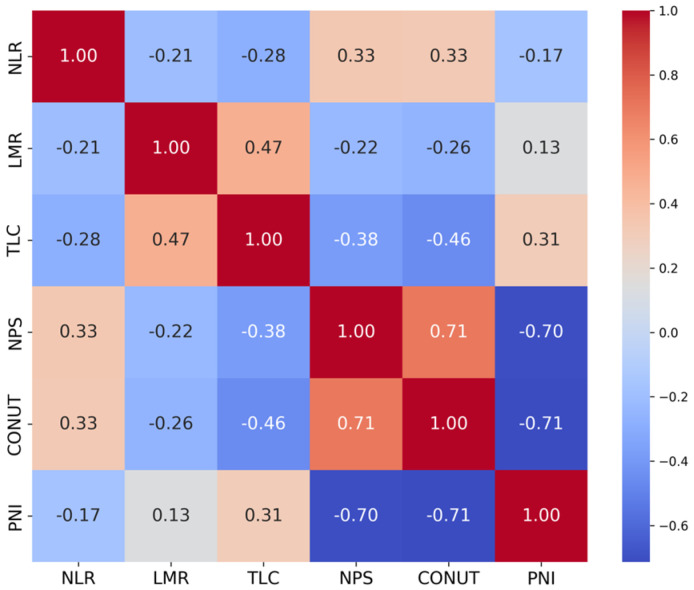
Correlation matrix of nutritional and inflammatory biomarkers included in the Cox proportional hazards model. Colors represent Pearson’s correlation coefficients (*r*), ranging from −1 to 1, with red indicating positive and blue indicating negative correlations. Diagonal elements correspond to r = 1, and numerical values within the cells denote the estimated correlation coefficients.

Variance inflation factors were below conservative thresholds (all < 5), indicating no material multicollinearity; the highest VIF was observed for CONUT (2.75). The correlation matrix showed the strongest pairwise relations for CONUT–NPS (r ≈ 0.71), CONUT–PNI (r ≈ −0.71), and NPS–PNI (r ≈ −0.70); all other coefficients were weak to moderate, and no |r| ≥ 0.8 was observed (Figure 2).

#### 3.3.3. Multivariable Model

After adjustment, two variables remained independently associated with overall survival: CONUT and LMR. Higher CONUT values were linked to an approximately 70% increase in the hazard of death, whereas higher LMR was associated with a lower hazard (protective effect). The remaining predictors lost statistical significance in the adjusted model (Table 3).

#### 3.3.4. Proportional Hazards and Time Dependency

Schoenfeld residuals indicated violation of the proportional hazards assumption for CONUT in the baseline model. Introducing a time-dependent term resolved this issue and confirmed the independent prognostic contribution of CONUT. The extended diagnostics also suggested non-proportionality for NLR and NPS, implying that their effects may vary over time. The final multivariable analysis identified CONUT as an independent adverse prognostic factor and LMR as an independent protective marker in patients receiving long-term home parenteral nutrition after gastrectomy.

### 3.4. ROC Analysis for LMR

**Figure 3:**
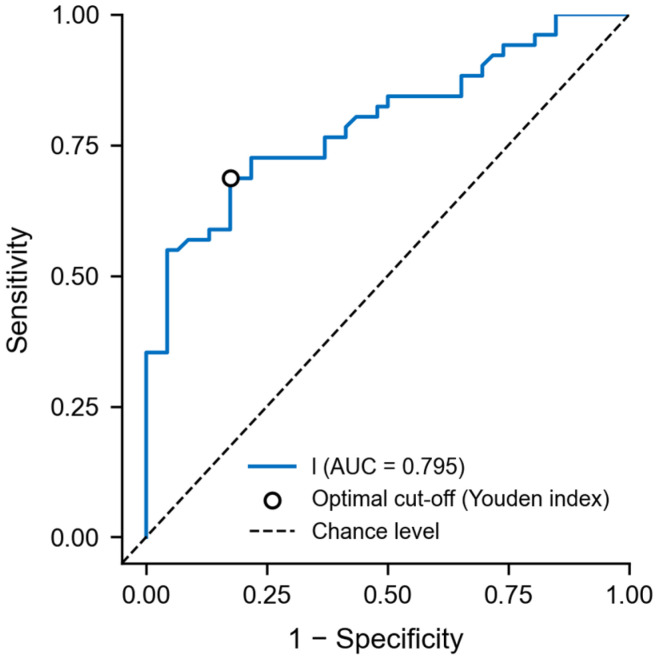
Receiver operating characteristic (ROC) curve of the lymphocyte-to-monocyte ratio (LMR) for predicting overall survival in patients with intestinal failure following gastrectomy. The area under the curve (AUC) was 0.795. The optimal cut-off value for LMR, identified using the Youden index, was 2.083, enabling stratification of patients into low- and high-LMR groups with respect to survival probability. The dashed line represents the reference line corresponding to random classification (AUC = 0.5).

The receiver operating characteristic curve for LMR showed moderate-to-good discrimination (AUC = 0.795). The Youden index identified an optimal cutoff of 2.083, partitioning patients into low (≤2.083) and high (>2.083) LMR groups (Figure 3).

### 3.5. Kaplan–Meier and Trend Analyses for CONUT and LMR

**Figure 4:**
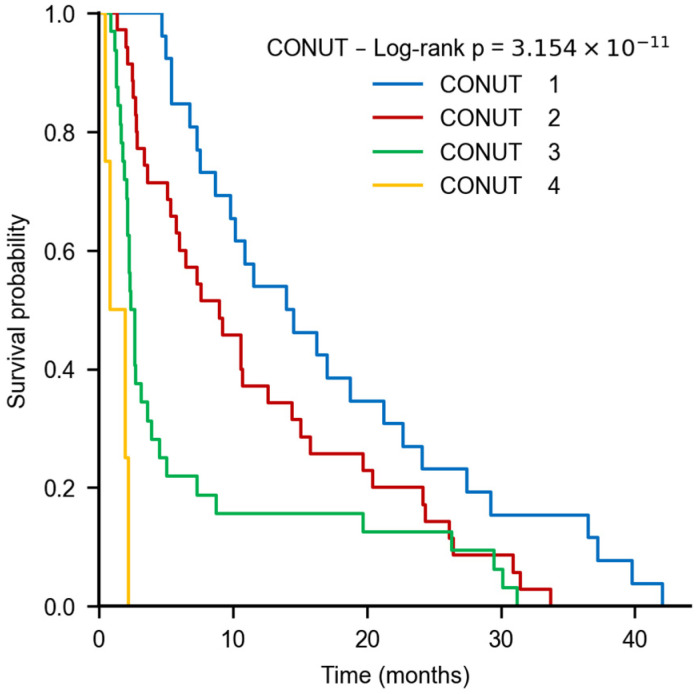
Kaplan–Meier survival curves stratified by CONUT categories. Patients were grouped according to CONUT scores (1–4). A stepwise and monotonic decline in overall survival was observed with increasing CONUT category, indicating progressively poorer prognosis with worsening nutritional status. Differences between groups were highly significant (log-rank test, *p* < 0.001).

For CONUT, Kaplan–Meier curves differed significantly across categories (global log-rank, *p* < 0.001). Holm-adjusted pairwise tests confirmed stepwise differences between adjacent categories (1 vs. 2, 2 vs. 3, 3 vs. 4; all significant). A Cox trend model treating CONUT as ordinal demonstrated a monotonic risk gradient: each one-category increase was associated with an approximately twofold higher hazard of death (HR ≈ 1.97; 95% CI, 1.50–2.58; *p* < 0.001) (Figure 4).

**Figure 5:**
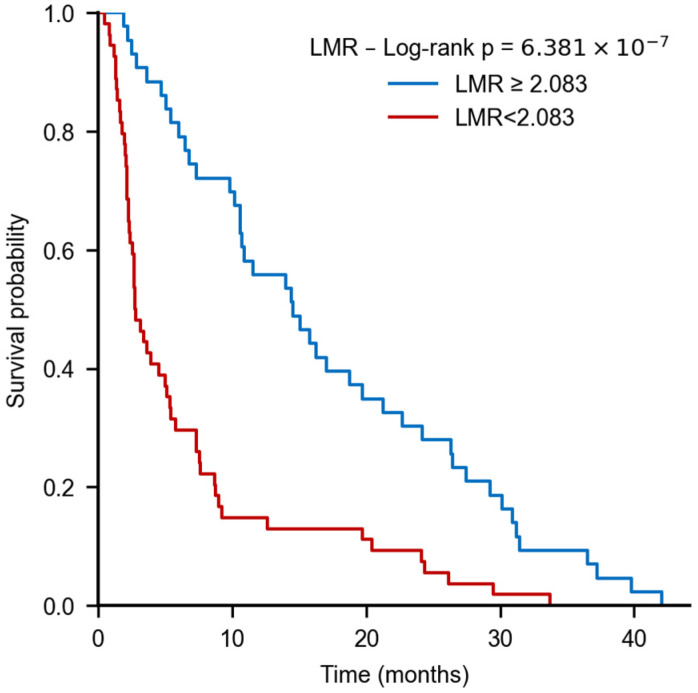
Kaplan–Meier survival curves for patients with low LMR. Patients with high LMR (>2.083) exhibited significantly longer overall survival compared with those with low LMR (≤2.083), with median survival times of 14.5 and 2.7 months, respectively (log-rank test, *p* < 0.001).

Using the LMR cutoff (2.083), Kaplan–Meier analysis showed a pronounced survival separation: median survival was 2.7 months in the low-LMR group (≤2.083) versus 14.5 months in the high-LMR group (>2.083), with a highly significant log-rank difference (*p* < 0.001) (Figure 5).

**Figure 6:**
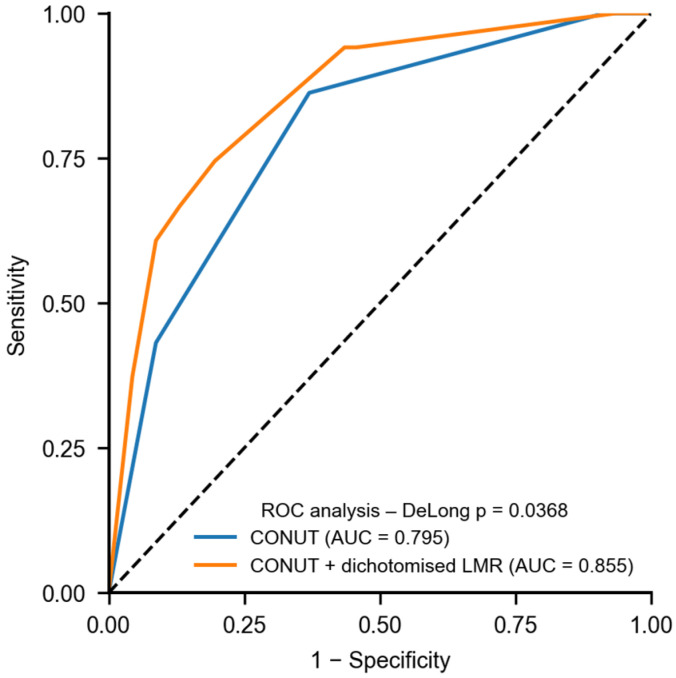
Receiver operating characteristic (ROC) curves comparing the prognostic performance of the CONUT score alone and the combined model including dichotomized lymphocyte-to-monocyte ratio (LMR_dich) for overall survival in patients with intestinal failure after gastrectomy. The area under the curve (AUC) was 0.795 for the CONUT score and 0.855 for the combined CONUT + LMR_dich model, indicating improved discriminatory ability when LMR_dich was incorporated. The dashed line represents the reference line corresponding to random classification (AUC = 0.5).

In the comparative analysis of predictive performance, the model incorporating both the CONUT index and the inflammatory marker LMR (model 2) demonstrated a higher AUC (0.855; 95% CI: 0.777–0.922) than the model including CONUT alone (AUC = 0.795; 95% CI: 0.709–0.874). This difference was statistically significant (DeLong test, *p* = 0.0368). Moreover, model 2 showed a significant improvement in patient classification, as reflected by the Net Reclassification Improvement (NRI = 1.025) and the Integrated Discrimination Improvement (IDI = 0.086), indicating a marked enhancement in distinguishing between patients with shorter versus longer survival (Figure 6).

Together, these results support CONUT and LMR as complementary prognostic indicators of nutritional and immune–inflammatory status in patients receiving long-term home parenteral nutrition after gastrectomy.

## 4. Discussion

The aim of this study was to evaluate the impact of nutritional and inflammatory parameters on survival in patients who, after gastrectomy for gastric cancer, developed chronic intestinal failure secondary to advanced malignancy. Our findings indicate that the composite immuno-nutritional indices CONUT and LMR are independent prognostic factors, underscoring the interplay between malnutrition, immunosuppression, and chronic inflammation in shaping outcomes in this highly vulnerable population.

Patients with chronic intestinal failure due to extensive cancer peritoneal spread represent a subgroup at exceptionally high metabolic risk [27]. In this setting, homeostasis depends on a careful balance between adequate energy provision and limitation of catabolic activity [28]. Inflammation and malnutrition are tightly interlinked in a self-reinforcing cycle: persistent inflammatory activity accelerates skeletal-muscle proteolysis [29], precipitating hypoproteinemia, hypoalbuminemia, and immune dysfunction [30,31], which in turn reduce tolerance to treatment and shorten overall survival [32]. In the present study, multivariable analysis demonstrated that CONUT and LMR were the only independent predictors of survival, indicating that a combined evaluation of nutritional status and inflammatory activity provides prognostic information beyond conventional anthropometric measures. Notably, BMI, despite its widespread use, was not associated with survival, likely because it does not capture changes in body composition or protein depletion characteristic of this population [33,34,35]. Skeletal muscle mass, commonly assessed using the Skeletal Muscle Index, represents an established prognostic marker in oncology [36]. Our findings further indicate that functional and immune-related parameters provide complementary prognostic information beyond body composition alone. BMI was originally conceived as a population-based measure of adiposity, which partly explains its limited prognostic utility at the individual level, particularly in patients with advanced cancer. Consistent with prior observations, patients with a normal BMI may still exhibit substantial protein–energy and micronutrient deficiencies [37].

These findings align with reports that malnutrition and chronic inflammatory activation are major determinants of prognosis in chronic metabolic and intestinal diseases, including intestinal failure [33]. In this clinical context, parenteral nutrition, although often indispensable, does not by itself guarantee improved outcomes; its effectiveness depends on the inflammatory burden, metabolic reserves, and regenerative capacity [38], as well as timely detection of malnutrition and initiation of targeted interventions [5].

Increasing attention has therefore focused on biochemical indices integrating nutritional and immune status, such as CONUT and PNI, that jointly reflect metabolic and immunological function [39,40]. The CONUT score incorporates markers of protein reserves and immune competence and has shown prognostic value across metabolic, cardiovascular, and malignant conditions [39,41]. Higher CONUT values have been linked to greater mortality risk, metabolic derangements, impaired wound healing, and lower effectiveness of nutritional therapy [42,43]; in surgical gastric-cancer cohorts, elevated preoperative CONUT has correlated with poorer outcomes despite adjuvant treatment [44]. In our analysis, a higher CONUT score remained an independent adverse factor (approximately 70% increase in mortality risk), supporting its relevance in routine monitoring and in decision-making regarding qualification for and management of parenteral nutrition.

Wang and colleagues were among the first to demonstrate that the preoperative CONUT score served as a significant prognostic indicator in patients undergoing surgical treatment, and that a CONUT-based predictive model outperformed PNI and NLR based approaches in estimating overall and recurrence-free survival. Although their study involved a different clinical population, namely patients with hilar cholangiocarcinoma, it provided important evidence that CONUT reflects meaningful immuno-nutritional disturbances with prognostic relevance [45]. More recently, Liu et al. demonstrated that preoperative immuno-nutritional status was significantly associated with the risk of postoperative complications in patients with ovarian cancer and could be incorporated into predictive models for postoperative risk stratification. Despite addressing a different malignancy and clinical endpoint, these findings further support the relevance of early immuno-nutritional assessment in surgically treated oncology patients as a component of clinical risk stratification [46]. In our cohort of post-gastrectomy patients with gastric cancer and chronic intestinal failure requiring HPN, we observed signals suggesting that CONUT may offer a prognostic advantage over the other assessed indices, implying that its predictive value may extend beyond the postoperative context and could remain clinically relevant in populations with markedly different disease trajectories [45].

LMR reflects the balance between cellular immunity and systemic inflammation. Low LMR denotes inflammatory predominance, immunosuppression, and greater susceptibility to infection [47]. Multicenter data have associated low LMR with poorer survival and reduced tolerance to therapy in chronic inflammatory conditions [14,41]. Our results are consistent with these observations and suggest that LMR is a sensitive, readily accessible indicator of clinical deterioration; its routine monitoring may help tailor the intensity of anti-inflammatory therapy, immunomodulatory supplementation, and metabolic support.

Although CONUT and LMR partially share input components and relate to overlapping pathophysiological domains, their independent prognostic value suggests that they capture distinct biological mechanisms. The absence of multicollinearity and only moderate correlation indicate that systemic inflammation and malnutrition, while interrelated, provide separate and complementary prognostic signals. This supports the combined use of nutritional and inflammatory markers to achieve more robust risk stratification in this clinical context. A previous study applied an immuno-nutritional framework to predict survival in patients undergoing adjuvant radiotherapy for cervical cancer. The investigators developed a prognostic nomogram combining the CONUT score with the platelet-to-lymphocyte ratio. Analyses based on Integrated Discrimination Improvement and Net Reclassification Improvement consistently demonstrated that this model provided superior overall survival prediction compared with the conventional FIGO staging system [48]. Our findings indicate that incorporating an inflammatory marker such as LMR into a model primarily based on nutritional assessment may substantially enhance its prognostic utility. Although CONUT alone exhibits solid predictive performance, the addition of information reflecting the magnitude of systemic inflammation allows for more refined risk stratification. Notably, both Integrated Discrimination Improvement and Net Reclassification Improvement metrics point to improved classification among patients after gastrectomy with chronic intestinal failure, regardless of whether their prognosis is favourable or unfavourable, suggesting potential relevance for personalized therapeutic decision-making.

This study has limitations. Its retrospective design and moderate sample size may restrict generalizability. Due to the retrospective design of the study and the rarity of the analysed population, a formal a priori power calculation was not performed. The use of a ROC-derived cutoff for LMR introduces information loss due to dichotomization. Nevertheless, the reliance on objective, quantitative laboratory parameters strengthen internal validity. The analysis was limited to baseline immuno-nutritional assessment and did not capture dynamic changes in immune parameters during oncological treatment. Detailed information on chemotherapy regimens and dosing was not analysed, as treatment decisions were made outside the study centre and were beyond the scope of this retrospective nutritional analysis. Finally, as the primary focus of the study was on the prognostic significance of the evaluated variables, we did not pursue a detailed analysis of potential time-varying effects. The inclusion of time-dependent terms served solely as a technical adjustment to ensure the validity of the Cox regression model.

To our knowledge, no previous studies have evaluated a combined CONUT–LMR model using reclassification metrics such as IDI and NRI. This underscores the novelty of our approach and suggests that combining a nutritional–immune index with an inflammation-based marker may offer an innovative and biologically grounded strategy for improving survival stratification in patients with malignant chronic intestinal failure after gastrectomy. Prospective, multicenter studies evaluating temporal variability and the dynamic trajectories of these indices are warranted to confirm the prognostic utility of CONUT and LMR and to assess their integration into composite predictive models that incorporate nutritional, inflammatory, and metabolic parameters. Such models may ultimately support the development of clinically applicable tools for risk stratification in malignancy-related intestinal failure.

## 5. Conclusions

This study demonstrates that routinely obtainable immuno-nutritional indices can assist in identifying patients with malignant chronic intestinal failure who are at increased risk of poor survival after gastrectomy. CONUT and LMR captured distinct prognostic dimensions and, when interpreted together, may help refine clinical assessment beyond standard measures.

These findings highlight the potential value of incorporating immuno-nutritional biomarkers into the routine evaluation of patients requiring long-term parenteral nutrition. Their integration may support earlier recognition of high-risk trajectories and guide individualized adjustments in nutritional and inflammatory management.

Future prospective, multicenter studies are needed to establish the temporal stability, clinical applicability, and predictive performance of combined models incorporating CONUT, LMR, and other metabolic markers. Such efforts may facilitate the development of pragmatic, biologically grounded tools to optimize care in malignancy-related intestinal failure.

## Figures and Tables

**Table 1:** Baseline nutritional and immuno-nutritional characteristics stratified by overall survival.

Variable	Survival < 176 Days (*n* = 46)	Survival ≥ 176 Days (*n* = 51)	*p* Value
NPS 0, *n* (%)	3 (6.5)	11 (21.6)	<0.01
NPS 1, *n* (%)	7 (15.2)	17 (33.3)	
NPS 2, *n* (%)	15 (32.6)	16 (31.4)	
NPS 3, *n* (%)	21 (45.7)	7 (13.7)	
CONUT 1, *n* (%)	4 (8.7)	22 (43.1)	<0.01
CONUT 2, *n* (%)	13 (28.3)	22 (43.1)	
CONUT 3, *n* (%)	25 (54.3)	7 (13.7)	
CONUT 4, *n* (%)	4 (8.7)	0	
BMI < 18.5, *n* (%)	27 (58.7)	23 (45.1)	0.401
18.5 ≤ BMI < 25, *n* (%)	16 (34.8)	23 (45.1)	
BMI ≥ 25, *n* (%)	3 (6.5)	5 (9.8)	
NLR, median (IQR)	3.88 (6.10)	2.04 (1.77)	<0.01
LMR, median (IQR)	1.54 (0.89)	2.84 (1.95)	<0.01
TLC, median (IQR)	745 (829.25)	1650 (934.50)	<0.01
PNI, median (IQR)	33.50 (8.75)	40.01 (7.51)	<0.01

Baseline laboratory parameters are presented for descriptive purposes and reflect the clinical status at the time of qualification for home parenteral nutrition. The stratification according to median overall survival was applied to facilitate the presentation of baseline characteristics and was not used for prognostic modelling. Data are presented as median (interquartile range [IQR]) or number (percentage), as appropriate. *p* values were calculated using the Mann–Whitney U test for continuous variables and the χ^2^ or Fisher exact test for categorical variables. Abbreviations: BMI, body mass index; CONUT, Controlling Nutritional Status; IQR, interquartile range; LMR, lymphocyte-to-monocyte ratio; NLR, neutrophil-to-lymphocyte ratio; NPS, Naples Prognostic Score; PNI, Prognostic Nutritional Index; TLC, total lymphocyte count.

**Table 2:** Baseline laboratory parameters stratified by overall survival time.

Variable	Survival < 176 Days (*n* = 46)	Survival ≥ 176 Days (*n* = 51)	*p* Value
Neutrophils, ×10^9^/L, median (IQR)	3 (6.5)	3.28 (2.17)	0.278
Lymphocytes, ×10^9^/L, median (IQR)	7 (15.2)	1.60 (0.85)	<0.01
Monocytes, ×10^9^/L, median (IQR)	15 (32.6)	0.57 (0.33)	0.096
Lymphocytes, %, median (IQR)	21 (45.7)	21.30 (16.30)	<0.01
WBC, ×10^9^/L, median (IQR)	4 (8.7)	7.00 (5.00)	0.853
Total cholesterol, g/dL, median (IQR)	13 (28.3)	166.0 (69)	0.128
Albumin, g/dL, median (IQR)	25 (54.3)	4.00 (0.75)	<0.01
Total protein, g/dL, median (IQR)	4 (8.7)	6.90 (0.70)	<0.01

Baseline laboratory parameters are presented for descriptive purposes and reflect the clinical status at the time of qualification for home parenteral nutrition. The stratification according to median overall survival was applied to facilitate the presentation of baseline characteristics and was not used for prognostic modelling. Data are presented as median (interquartile range [IQR]). *p* values were calculated using the Mann–Whitney U test. Abbreviations: IQR, interquartile range; WBC, white blood cells.

**Table 3:** Univariate and multivariate Cox regression analysis for selected nutritional and inflammatory indices for overall survival.

Variables	Univariate Analysis			Multivariate Analysis		
		HR (95% CI)			HR (95% CI)	
	HR	Lower–Upper	*p*-Value	HR	Lower–Upper	*p*-Value
Controlling Nutritional Status	1.969	1.503–2.580	<0.01	1.724	1.116–2.663	<0.01
Prognostic Nutritional Index	0.934	0.902–0.967	<0.01	0.958	0.911–1.007	0.10
Lymphocyte-to-Monocyte	0.762	0.653–0.890	<0.01	0.793	0.680–0.925	<0.01
Naples Prognostic Score	1.433	1.164–1.764	<0.01	0.860	0.635–1.166	0.334
Neutrophil-to-Lymphocyte Ratio	1.024	1.008–1.040	<0.01	1.009	0.990–1.028	0.349
Total Lymphocyte Count	0.9997	0.999–0.999	<0.01	1.000	0.9999–1.0003	0.411
Body Mass Index	0.980	0.927–1.035	0.46	-	-	-

Data are presented as hazard ratios (HRs) with 95% confidence intervals (CIs). *p* values were calculated using Cox proportional hazards regression models. Abbreviations: CI, confidence interval; HR, hazard ratio.

## Data Availability

The data presented in this study are available on request from the corresponding author for any academic use upon citation of this article. The data are not publicly available due to privacy and permission constraints that restrict their use to the publication of this article only.

## Abbreviations

The following abbreviations are used in this manuscript:

AUC	Area under the receiver operating characteristic curve
BMI	Body Mass Index
CONUT	Controlling Nutritional Status
HR	Hazard Ratio
HPN	Home Parenteral Nutrition
IDI	Integrated Discrimination Improvement
LMR	Lymphocyte-to-Monocyte Ratio
NLR	Neutrophil-to-Lymphocyte Ratio
NPS	Naples Prognostic Score
NRI	Net Reclassification Improvement
PNI	Prognostic Nutritional Index
ROC	Receiver Operating Characteristic Curve
TLC	Total Lymphocyte Count
VIF	Variance Inflation Factor
